# Supplementary material for: New clusterization of global seaport countries based on their DEA and FDEA network efficiency scores
Source: PLoS One. 2024 Jul 30;19(7):e0305146. doi: 10.1371/journal.pone.0305146 (PMC11288466; doi:10.1371/journal.pone.0305146)
Supplement: Attachment — Submitted filename: Response to Reviewers.pdf [file pone.0305146.s001.pdf]

Response to Reviewers

PONE-D-23-40899 : New Clusterization of Global Seaport Countries Based on Their DEA and FDEA Network Efficiency Scores

Dear Respected Editor and Reviewers,

First of all, we would like to thank all of you for giving our manuscript the opportunity to be reviewed for possible publication in the journal of PLOS ONE.

We have improved the language and the contents in the revised manuscript as per requests by the respected reviewers.

Reviewer 1

Dear authors,
The work presents k-means and hierarchical approaches, using the results of Data Envelopment Analysis and Fuzzy Data Envelopment Analysis to aggregate 133 countries according to the efficiency scores of their port networks. The study approach makes interesting contributions to the topic and in this regard, thank you for the effort and dedication of the authors. However, some issues must be considered to provide a better understanding. Recommendations for the manuscript are as follows:

Observation 1: Abstract
The abstract provides a contextualization of the subject, some results and part of the conclusion, but does not highlight the gap explored.
Authors' Reply: We have added the gap explored in this study in the abstract as suggested by the reviewer (Line 4-8).

Observation 2: Number the sections
At the end of the introduction, the organization of the manuscript into numbered sections is presented, which does not match the sequence of the text. On page 14 there is a number: "4.5.2. Hierarchical versus Hkmeans for DEA and FDEA".
Authors' Reply: We are sorry for the inconsistence in the section numbering. We now have add the section numbering to match the sequence on the text at the end of the introduction.

Observation 3: Improve the Literature Review
The manuscript presents an interesting review, however, this section could be written in a more concise way, and some sentences could be improved to make the reading flow smoother. Connections with references can be presented in a more direct way; for example, instead of "a study" the authors could already carry out the citation as done in some parts of the text. In particular, paragraph 4 could be rewritten in a way that better relates the sentences. Furthermore, some contributions highlighted at the end of this section can be used in the introduction or conclusion.

Authors’ Reply: Thank you for the comment and suggestion. We have improved the connection between sentences and references to be in a more direct way by mentioning the respective author names under the Literature Review section - Paragraph 3 and Paragraph 4.

Observation 4: Standardization figures

Some figures are different and on page 13 the x-axis title overlaps the content.

Authors’ Reply: The clustering figures in this manuscript are presented as follows:

a) Figures 6 to 8 are focusing on hierarchical clustering for 2018, 2019 and 2020.
b) Figure 9 highlight hierarchical k-means clustering for DEA and FDEA datasets
c) Figure 11 and 12 demonstrate hierarchical and hkmeans clustering side by side

More explanations on the colour notation of the dendogram branches and country number have been added in Lines 5-10, Paragraph 1 of Section 4.3 and in Lines 1-14, Paragraph 2 of Section 4.4. Moreover, the technical error where the x-axis title overlaps the content of Figure 9 has been fixed.

Observation 5: Conclusion

The conclusion is too lengthy; the authors can only highlight the main findings of the study and the limitations.

Authors’ Reply: We have revised the conclusion by highlighting the main findings, limitation and future study (Reviewer 2) as suggested while reducing the length of the conclusion part from originally 786 words to presently 397 words.

Reviewer 2:

The design of this study is commendable, data organization and analysis are well done, but the discussion section needs to be improved and mined deeply. Some questions are as below.

1. The abstract is too long and not easy to follow. Moreover, the expression "it is the first literature that" is not appropriate and requires caution.
Authors' Reply: We have shorten the abstract from originally 250 words to 216 words presently. Moreover, we have removed the expression "it is the first literature that" from the revised abstract.

2. The authors should state the motives of their study and tell readers why their study is important and useful to academics and practitioners.
Authors' Reply: Thank you for the comment and suggestion. We have added the motivation and the importance of the study that will be useful for academics and practitioners in Line 1-18, Paragraph 5 (last Paragraph) of Section 1.

3. The authors should strengthen their contribution and clarify what is really their contribution and what has already been done in the literature. This is very important and in the current form of the paper their contribution seems very limited.
Authors' Reply: The contributions of the study have been strengthened and clarified in the last Paragraph of Section 2 as follows:

1) This study introduces four new level clusters specified as low connectivity (LC), medium connectivity (MC), high connectivity (HC) and very high connectivity (VHC) in clustering the seaport network efficiency scores. The nearest study by Andrade et al. [20] categorized leading Brazilian ports based on cargo throughput efficiency, dividing them into only three groups (highly efficient, moderately efficient and inefficient).
2) This study utilizes DEA and FDEA in the countries' seaport network efficiency measurement prior to the conduct of clustering. None of the previous studies including [21] - [31] have considered both DEA and FDEA in the clustering strategy applications at all.
3) This study clusters 133 global seaport countries which is the highest number of countries considered in similar research area. Previously Nguyen and Woo [16], employed k-means cluster analysis for top 10 Southeast Asian ports' countries.
4) This study applies k-means and hierarchical strategies as well as recommends the third strategy, hkmeans (hierarchical k-means) for seaport network efficiency clustering. In the nearest past study, k-means strategy has been applied in social network analysis within the maritime transportation context, focusing on the top 10 Southeast Asian ports [16].
5) The present study implements LSCI and GDP output in both DEA and FDEA computations prior to the clustering application which was never been carried out before. Previously, Chang et al., [11] has only used LSCI to perform hierarchical clustering strategy.

4. Methodology: The author needs to articulate the reasons for using FDEA and its advantages, what problems the method solves. The methods are existing methods. I suggest authors provide explanations on what and why the methodology was applied in this study. Please provide the advantages of your model.
Authors' Reply: We have added the reason and the advantages of FDEA as well as the problems that it can solve in the Last Paragraph of Section 3.1.

5. For the seaport network efficiency evaluation in DEA models, the author should consider some undesirable outputs.
Authors' Reply: Thank you for the comment and suggestion. We have highlighted the undesirable outputs as follows:

a) Line 5-9, Paragraph 4 of Section 4.2
b) Line 26-39, Paragraph 1 of Section 4.3.

6. The author describes many kinds of Clustering methods, but the applicability of these kinds of Clustering are all different and cannot be used together for comparison.
Authors' Reply: We have describe the different kinds of clustering methods considered in this study together with their application in the First Paragraph of Section 4.5.2.

7. The dissection of empirical results needs to be supplemented with more profound insights. For example, economic realities corresponding to the results should be found to corroborate them.
Authors' Reply: We have added the profound insights of empirical results as suggested by the reviewer in the entire Paragraph 10 of Section 4.5.3.

8. Some graphical displays cannot intuitively reveal the results, such as Figure 9, Figure 12, and so on.
Authors' Reply: The clustering figures in this manuscript are presented as follows:

a) Figures 6 to 8 are focusing on hierarchical clustering for 2018, 2019 and 2020.
b) Figure 9 highlight hierarchical k-means clustering for DEA and FDEA datasets
c) Figure 11 and 12 demonstrate hierarchical and hkmeans clustering side by side

More explanations on the colour notation of the dendogram branches and country number have been added in Lines 6-10, Paragraph 1 of Section 4.3 and in Lines 1-14, Paragraph 2 of Section 4.4.

9. The author should also supplement the limitations of this study and suggestions for future research in the conclusion section.
Authors' Reply: We have revised the conclusion by highlighting the main findings (Reviewer 1), limitation and future study as suggested.
